# The Risk of New-Onset Atrial Fibrillation in Patients With Conduction System Pacing Versus Right Ventricular Pacing: A Meta-Analysis

**DOI:** 10.31083/RCM27921

**Published:** 2025-04-18

**Authors:** Tingwen Gao, Zhaofeng Li, Wei Li, Xue Wang, Xinxing Xie

**Affiliations:** ^1^Department of Cardiology, Rizhao Heart Hospital Qingdao University, 276800 Rizhao, Shandong, China

**Keywords:** atrial fibrillation, His bundle pacing, left bundle branch pacing, right ventricular pacing, meta-analysis

## Abstract

**Background:**

Prior studies have established the safety and efficacy of conduction system pacing (CSP) in improving echocardiographic parameters and clinical outcomes. This meta-analysis aimed to investigate whether CSP could reduce the occurrence of new-onset atrial fibrillation (AF) in comparison to traditional right ventricular pacing (RVP) therapy.

**Methods:**

A literature search was performed in PubMed, Embase, and the Cochrane Library to identify relevant clinical studies comparing CSP with RVP from January 2000 to June 2024. The study outcome was new-onset AF after pacemaker implantation. Estimated risk ratios (RR), odds ratio (OR) with 95% confidence intervals (CI) were evaluated.

**Results:**

Our analysis included 8 observational studies comprising a total of 2033 patients. The results indicated that 20% (406/2033) of study patients experienced new-onset AF, and CSP was associated with a significantly lower risk of new-onset AF when compared with RVP (RR: 0.44, 95% CI: 0.36–0.54, *p* < 0.00001, I^2^ = 11%; OR: 0.34, 95% CI: 0.27–0.44, *p* < 0.0001, I^2^ = 0). In the subgroup analysis, patients with atrioventricular block (AVB) tended to benefit more from CSP than those with sinus node dysfunction (SND) or AVB (*p* = 0.06 for RR; *p *= 0.12 for OR). Publication bias was observed and confirmed by the Egger's test (*p *= 0.0125 for RR and 0.0345 for OR). Trim and fill analysis was performed, and the overall summary effect size (RR: 0.51, 95% CI: 0.40–0.64; OR: 0.40, 95% CI: 0.31–0.52) remained significant after adjusting for publication bias.

**Conclusion:**

CSP could reduce the occurrence of new-onset AF compared with RVP, and this benefit appeared to be more pronounced in patients with AVB than those with SND or AVB. However, large scale randomized controlled trials are needed to validate our findings.

**The PROSPERO Registration:**

Registration number: CRD42024569052; registration date: July 25, 2024; https://www.crd.york.ac.uk/PROSPERO/view/CRD42024569052.

## 1. Introduction

Cardiac pacing is usually indicated in patients with symptomatic bradycardia including sinus node dysfunction (SND) and atrioventricular conduction disorders to improve quality of life and life expectancy [1]. However, chronic right ventricular (RV) pacing (RVP), whether from the RV apex or septum, is correlated to increased risk of new-onset atrial fibrillation (AF) and heart failure (HF) hospitalization, especially in patients with a high ventricular pacing (VP) burden [2, 3, 4]. Long-term RVP could induce electromechanical desynchrony, which in turn resulted in the enlargement and decreased function of the left atrium (LA) [5, 6]. This atrial remodeling might theoretically contribute to the occurrence of atrial arrhythmias. Thus, alternative pacing sites are being explored for a better clinical outcome.

Currently, conduction system pacing (CSP), which includes His bundle pacing (HBP) and left bundle branch pacing (LBBP), has been recognized as a potential alternative pacing strategy in clinical practice because it restores or preserves the ventricular activation by stimulating the His-Purkinje system directly. Massive studies have proven the safety and efficacy of CSP in a wide range of patients in improving echocardiographic parameters and clinical outcomes [7, 8]. In a retrospective single-center study that composed of 477 patients, Pastore *et al*. [3] found that HBP exhibited a lower incidence of persistent/permanent AF when compared with RVP. Subsequently, more studies focused on the risk of AF after CSP [9, 10, 11, 12], and one meta-analysis reported a reduced risk of new-onset AF in LBBP group compared with RVP group [13]. Considering the recently published studies comparing the risk of new-onset AF between CSP and RVP [14, 15], an updated meta-analysis was needed to combine previous and current evidence for comprehensively assessing the association between CSP and new-onset AF. Therefore, this study aimed to evaluate the impact of CSP on new-onset AF in comparison to conventional RVP in patients without AF history.

## 2. Methods

This meta-analysis was performed following the Preferred Reporting Items for Systematic Reviews and Meta-Analyses (PRISMA) statement [16] and registered at the Prospective International Register of Systematic Reviews (PROSPERO, registration number CRD42024569052; registration date: July 25, 2024; https://www.crd.york.ac.uk/PROSPERO/view/CRD42024569052).

### 2.1 Search Strategy

A literature search utilizing PubMed, Embase, and the Cochrane library was conducted to retrieve all relevant studies from January 2000 to June 2024, because permanent HBP in human was first reported in 2000 by Deshmukh *et al*. [17]. The following search terms were used: “His bundle pacing, left bundle branch pacing, left bundle branch area pacing, right ventricular pacing, atrial fibrillation, atrial high rate episode”. Furthermore, the references of all included studies were also assessed to identify possibly relevant studies.

### 2.2 Study Selection

Two independent reviewers (TG and ZL) assessed the retrieved citations for inclusion and any controversies were resolved through discussion with a third investigator (XX). A study was enrolled in the analysis based on the following criteria: (1) randomized controlled trials (RCTs) and observational studies which directly compared the effects of CSP (HBP or LBBP) with RVP in bradyarrhythmia patients indicated for de novo pacemaker (PM) implantation, and (2) the study reported the occurrence of AF in each group, and (3) published in English, and (4) the minimum follow-up time >6 months, (5) conference abstracts and letters were also included if they met the four aforementioned criteria. We excluded: (1) editorials, reviews, case reports and meta-analyses, and (2) the study included patients with prior AF history but the new-onset AF incidence could not be distinguished in patients without AF history.

### 2.3 Study Outcomes

The primary outcome was the new-onset AF including clinical AF or sub-clinical AF (SCAF) defined as atrial high rate episodes (AHREs) detected by PM.

### 2.4 Data Collection and Quality Assessment

Two independent investigators (TG and ZL) extracted pertinent data employing a predefined data extraction form, which was subsequently verified by another two authors (WL and XW). Data regarding characteristics of the study, patients’ baseline characteristics, pacing model, ventricular pacing burden, the incidence of new-onset AF, and follow-up were extracted and collected. The quality of the observational studies was evaluated by two authors (XX and TG) utilizing the Newcastle-Ottawa Scale (NOS).

### 2.5 Statistical Analysis

Risk ratio (RR), odds ratio (OR) with 95% confidence interval (CI) were calculated as summary estimates. Heterogeneity was assessed using the Cochran’s Q test with a significance level at *p* < 0.10. Also, the Higgins I-squared (I^2^) statistic was used to test heterogeneity, and I^2^ values <25%, 25%–49%, and ≥50% were considered low, moderate, and high degrees of heterogeneity, respectively. A fixed-effects model was utilized for meta-analysis if there was no significant heterogeneity among the studies. Otherwise, a random-effects model was utilized. The sensitivity analysis was conducted to identify the source of heterogeneity using the leave-one-out method. Publication bias was evaluated by funnel plot analysis and Egger’s test, and a ‘trim and fill’ approach was utilized to correct it if there was a publication bias [18]. Subgroup analyses were performed according to study design, pacing mode, pacing indication, follow-up and sample size. Revman 5.4 (the Cochrane Collaboration, Copenhagen, Denmark) and Stata Software (version 17, Statacorp, College Station, TX, USA) were used for all statistical analyses.

## 3. Results

### 3.1 Baseline Characteristics

**Fig. 1.**
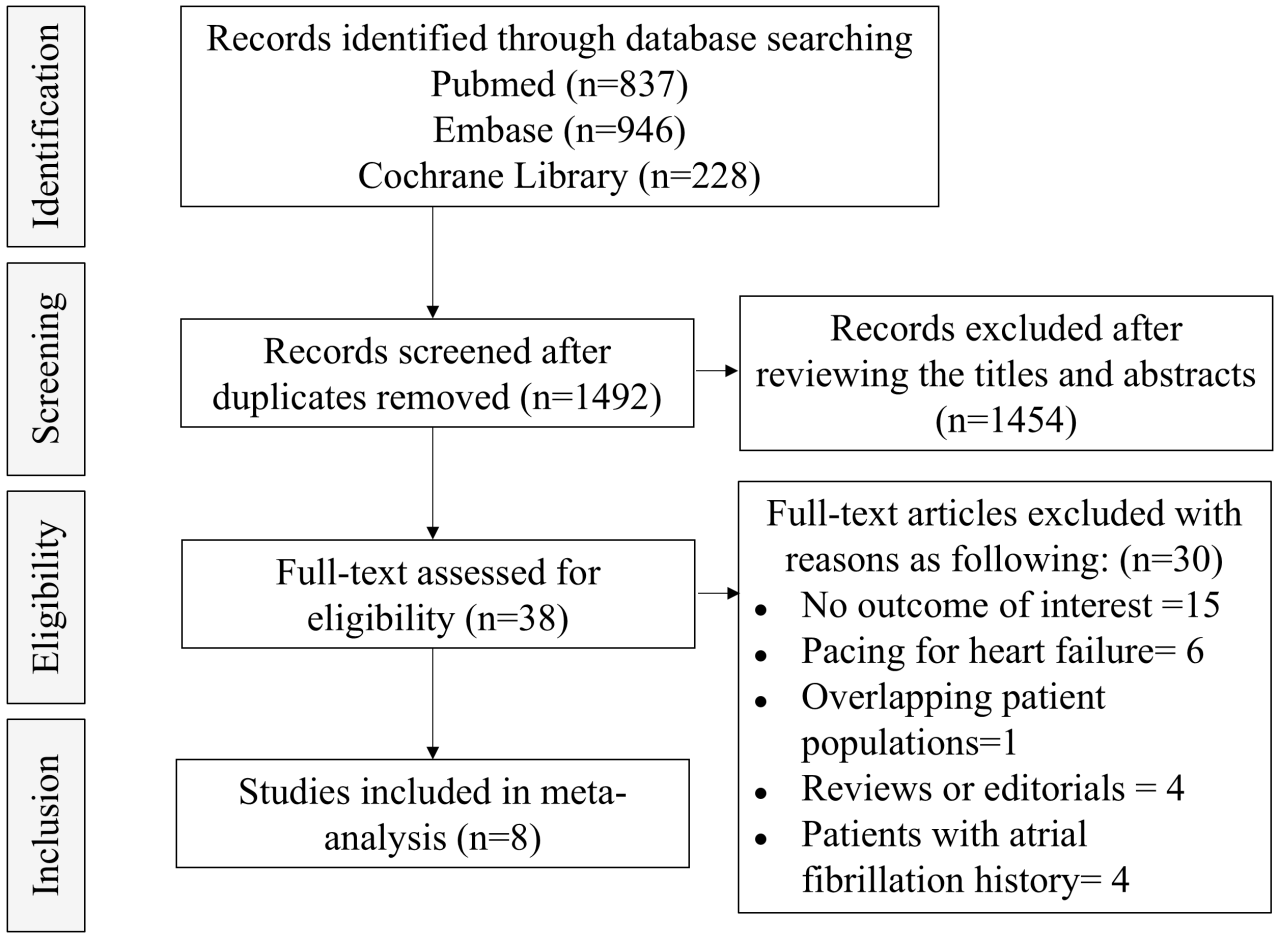
**Flow diagram for study selection**.

As presented in Fig. 1, the initial search generated 1492 citations after deduplication, of which 38 were deemed to be potentially eligible for inclusion. Ultimately, 8 observational studies were enrolled in our analysis comprising a total of 2033 patients. The quality of all eligible studies was assessed with high NOS scores as shown in Table 1 (Ref. [3, 9, 10, 11, 12, 14, 15, 19]).

**Table 1.** **Quality assessment of the included studies by using the Newcastle-Ottawa Scale**.

Criteria	Pastore G 2016 [3]	Ravi V 2020 [10]	Ravi V 2022 [11]	Zhu HJ 2023 [9]	Yang WY 2024 [14]	Zhang SG 2024 [15]	Takahashi M 2024 [12]	Ramos-Maqueda J 2024 [19]
Representativeness of the exposed cohort	1	1	1	1	1	1	1	1
Selection of the non-exposed cohort	1	1	1	1	1	1	1	1
Ascertainment of exposure	1	1	1	1	1	1	1	1
Outcome of interest was not present at the start	1	1	1	1	1	1	1	1
Comparability	2	2	2	2	2	2	2	1
Assessment of outcome	1	1	1	1	1	1	1	1
Enough follow-up	1	1	1	1	1	1	1	1
Adequacy of follow-up	1	1	1	1	1	1	1	0
Total	9	9	9	9	9	9	9	7
Quality	High	High	High	High	High	High	High	High

Of these 8 studies, 3 compared HBP with RVP (203 vs 397 patients) [3, 10, 12] and 5 evaluated the occurrence rate of new-onset AF between LBBP and RVP (679 vs 754 patients) [9, 11, 14, 15, 19]. Three were prospectively designed and the remaining 5 were retrospective. There were 6 original articles, one letter [11], and one abstract [19]. The pacing indication included SND or atrioventricular block (AVB) in 3 studies [9, 10, 19] and exclusively AVB in 4 studies [3, 12, 14, 15]. Four studies [3, 12, 14, 15] with a pacing indication of AVB reported a significantly high VP percentage (VP%, 85%~100%). As for the definition of new-onset AF, the majority of the studies (at least 7 out of 8 studies) confirmed the diagnosis of AF based on PM-detected AHREs which usually were manually checked. Three studies [9, 10, 11] reported the occurrence rate of new-onset AF in different subgroups categorized by VP%, which suggested that patients with VP% ≥20% could benefit more from CSP therapy in terms of a lower occurrence of new-onset AF. The main baseline characteristics of enrolled studies and patients are presented in Table 2 (Ref. [3, 9, 10, 11, 12, 14, 15, 19]).

**Table 2.** **Baseline characteristics of included studies in this meta-analysis**.

Study	Study design	Pacing mode	Sample size	Indication for pacing	Age (years)	LVEF (%)	LAD (mm)	VP burden (%)	FU (months)	Outcomes of atrial fibrillation
Pastore G 2016 [3]	retrospective	HBP	148	AVB	74.1 ± 8.5	62 ± 7	47.6 ± 8.1	100%	67.2 ± 28.7	(1) The ﬁrst occurrence of persistent or permanent AF in patients without a prior AF history; (2) The ﬁrst occurrence of progression to persistent or permanent AF in patients with previous AF event before PM implantation
		RVS or RVA	140		76.9 ± 7.0	60 ± 8	46.5 ± 7.8		53.2 ± 23.7	
			189		79.1 ± 8.3	60 ± 7	48.2 ± 8.1		55.2 ± 27.2	
Ravi V 2020 [10]	retrospective	HBP	105	SND or AVB	72.65 ± 11.04	59.84 ± 8.06	NA	NA	23.4 ± 10.8	(1) New onset AF among patients without a known history of AF; (2) Progression of AF defined as an absolute increase in average daily AF burden by ≥10% from the AF burden at initial device follow-up
		RVP	120		76.54 ± 9.87	61 ± 7.19				
Ravi V 2022 [11]	retrospective	LBBAP	173	NA	NA	NA	NA	NA	20 ± 9.3	New-onset AF episodes ≥30 seconds or ≥6 minutes detected on scheduled device follow-up performed in-person and remotely
		RVP	237							
Zhu HJ 2023 [9]	prospective	LBBAP	257	SND or AVB	63.6 ± 13.5	62.8 ± 4.9	36.9 ± 5.6	NA	11.1 ± 7.5	New-onset AF was defined as device-detected AF episodes lasting at least 30 s on intracardiac electrogram or surface 12-lead ECG. AHREs (atrial rate ≥190 bpm) detected by devices should be manually checked
		RVP	270		66.9 ± 11.5	63.1 ± 5.4	37.5 ± 5.9			
Yang WY 2024 [14]	retrospective	LBBAP	16	AVB	68.19 ± 14.77	67.31 ± 7.02	37.61 ± 4.23	>85	37.9	AHREs were defined as events with an atrial frequency of ≥176 bpm lasting for ≥6 minutes recorded by PM during follow-up. AHREs should be manually checked
		RVS or RVA	13		75.08 ± 9.01	64.92 ± 3.37	40.1 ± 5.94		30	
			11		67.64 ± 15.49	62.39 ± 8.89	39.76 ± 6.59		28.6	
Zhang SG 2024 [15]	prospective	LBBAP	43	AVB	75.0 ± 10.6	62.2 ± 2.5	38.6 ± 5.0	99.6 ± 1.0	14.1 ± 7.5	AHREs were defined as events with an atrial frequency of ≥175 bpm and a duration of ≥5 min detected by a PM device. AHREs should be manually checked
		RVP	43		72.4 ± 10.0	63.3 ± 5.2	38.1 ± 4.5	88.1 ± 20.9		
Takahashi M 2024 [12]	retrospective	HBP	22	AVB	79 ± 8	68.5 ± 3.6	39.4 ± 6.7	99 ± 2	21.1 ± 6	New-onset AHREs was defined as AHREs which occurred more than 3 months after PM implantation and lasted for >6 minutes at an atrial heart rate >190 bpm
		RVP	47		78 ± 8	66.7 ± 5.0	36.5 ± 6.3	97 ± 10	16.8 ± 8	
Ramos-Maqueda J 2024 [19]	prospective	LBBAP	198	SND or AVB	81.8 ± 6.4	58.4 ± 5.9	NA	NA	24	New-onset AF was defined as device-detected AF at least 6 minutes on intracardiac electrocardiogram or surface ECG
		RVS	193		82.3 ± 7	59.4 ± 6				

Abbreviations: AF, atrial fibrillation; AHREs, atrial high rate episodes; AVB, atrioventricular block; ECG, electrogram; FU, follow-up; HBP, His bundle pacing; LAD, left atrium diameter; LBBAP, left bundle branch area pacing; LVEF, left ventricular ejection fraction; NA, not available; PM, pacemaker; RVA, right ventricular apex; RVP, right ventricular pacing; RVS, right ventricular septum; SND, sinus node dysfunction; VP, ventricular pacing.

### 3.2 Results from Meta-analysis

**Fig. 2.**
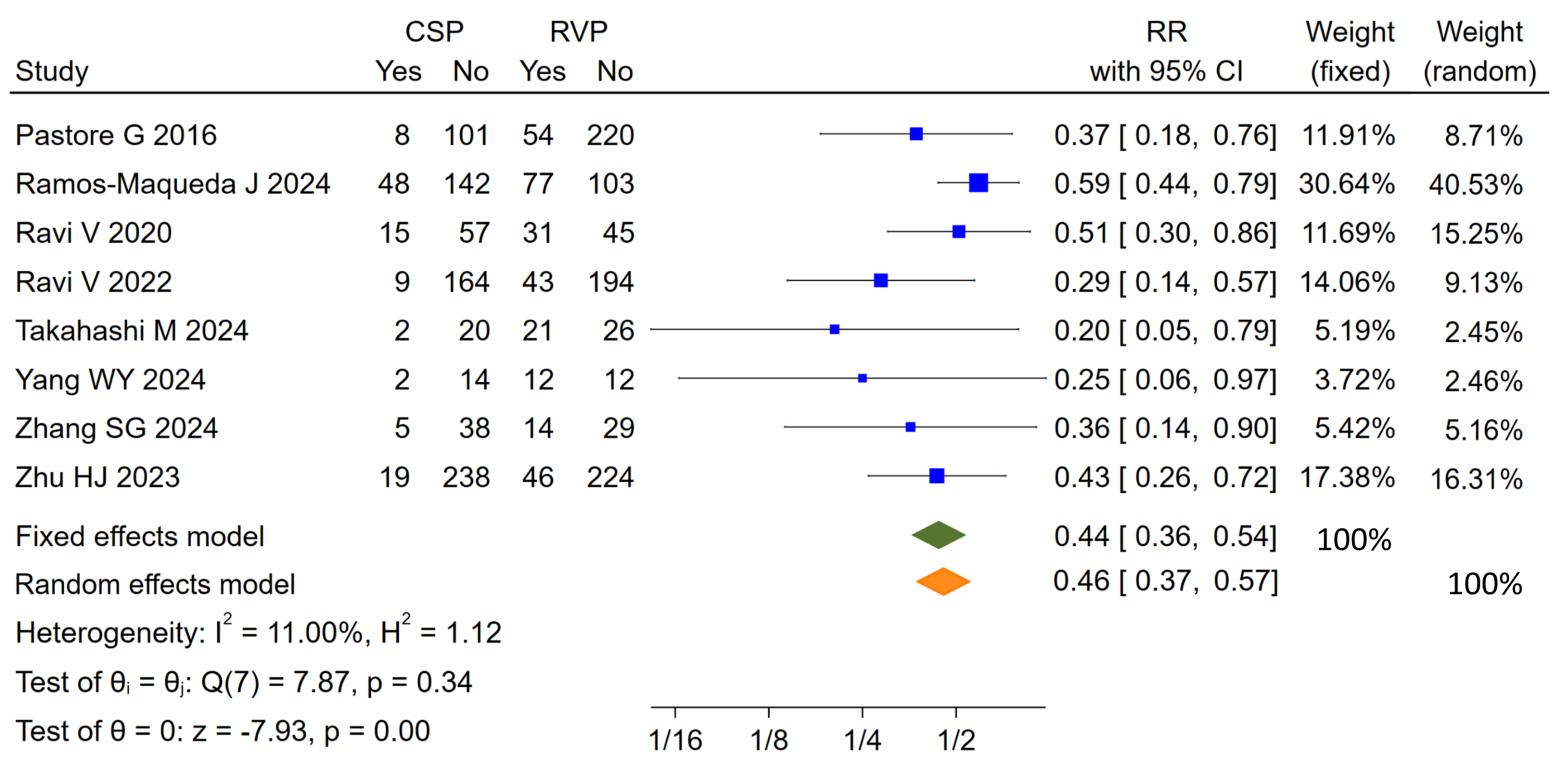
**Forest plot of pooled results for new-onset AF between CSP and RVP group**. AF, atrial fibrillation; CSP, conduction system pacing; RVP, right ventricular pacing; RR, risk ratios; CI, confidence interval.

**Fig. 3.**
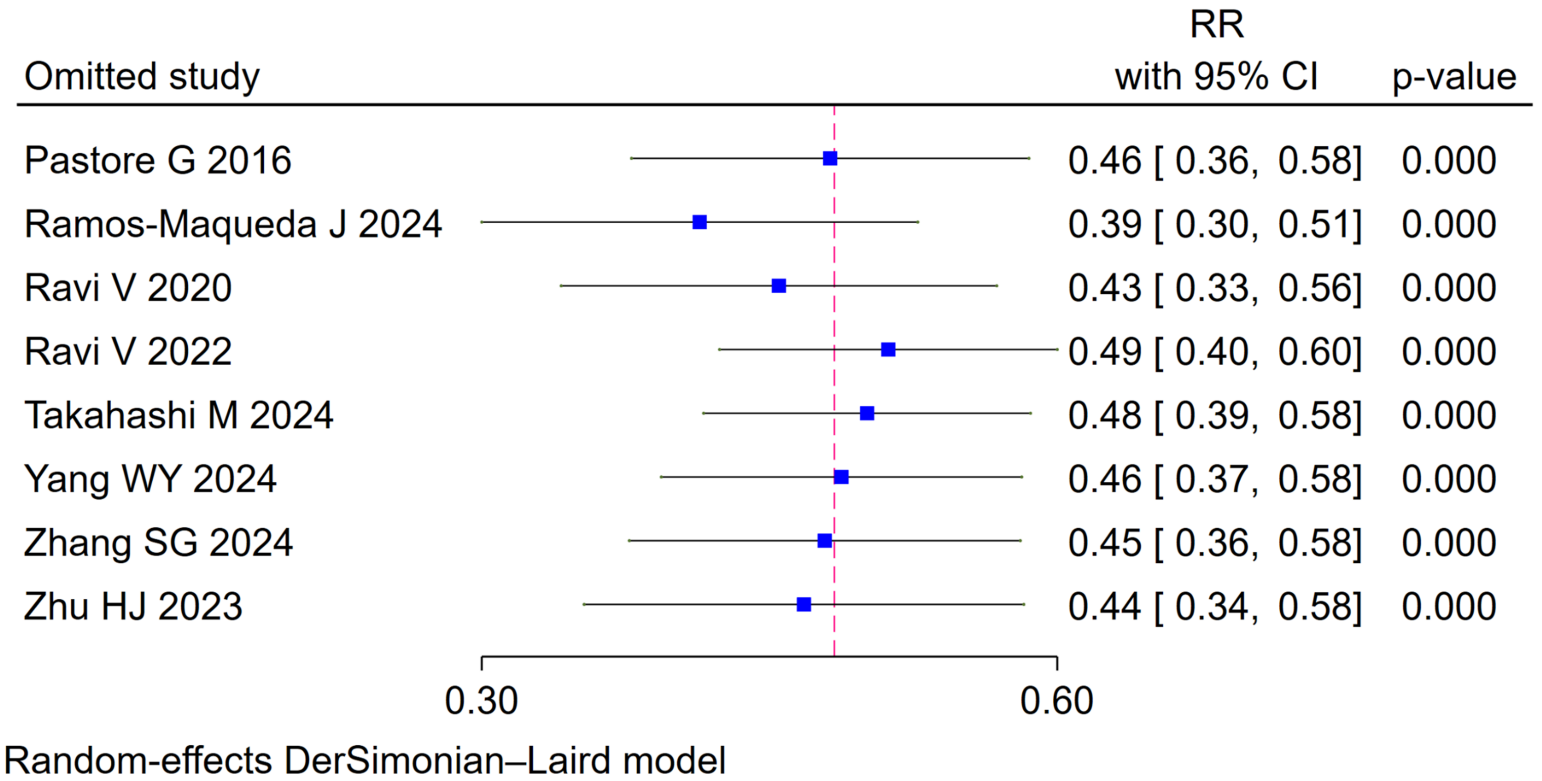
**Sensitivity analysis**. RR, risk ratio; CI, confidence interval.

Pooled analysis indicated that new-onset AF developed in 20% (406/2033) of all patients, and a lower incidence of new-onset AF was observed in the CSP group when compared with the RVP group (12.2% vs 25.9%). CSP was correlated to a significantly reduced risk of new-onset AF when compared with RVP whether using fixed effects model (RR: 0.44, 95% CI: 0.36–0.54, *p* < 0.00001; OR: 0.34, 95% CI: 0.27–0.44, *p* < 0.0001) or random effects model (RR: 0.46, 95% CI: 0.37–0.57, *p* < 0.0001; OR: 0.35, 95% CI: 0.27–0.46, *p* < 0.0001), and no substantial heterogeneity was found (*p* = 0.34–0.62, I^2^ = 0–11%) (Fig. 2 and **Supplementary Fig. 1**). Whether using random-effects models or fixed effects model with conservative 95% CI adjustment, the omission of each trial at a time showed a consistent result favoring CSP in reducing the occurrence rate of new-onset AF compared with RVP (Fig. 3 and **Supplementary Fig. 2**).

Subgroup analysis based on study design, pacing mode, pacing indication, follow-up and sample size was performed. As illustrated in Table 3, no significant difference was observed between the CSP and RVP groups in all the subgroup analyses, but the performance of CSP appeared more pronounced than RVP in patients with a pacing indication of AVB with a tendency to reduce the occurrence of new-onset AF (*p* = 0.06 for RR; *p *= 0.12 for OR).

**Table 3.** **Subgroup analysis**.

A							
Subgroup		Number of included studies	CSP	RVP	RR (95% CI)	*p*	Group difference
Study design	Prospective studies	3	72/490	137/493	0.53 [0.41, 0.68]	<0.00001	0.10
	Retrospective studies	5	36/392	161/658	0.37 [0.27, 0.52]	<0.00001	
Pacing mode	HBP	3	25/203	106/397	0.40 [0.26, 0.60]	<0.0001	0.57
	LBBP	5	83/679	192/754	0.46 [0.36, 0.58]	<0.00001	
Pacing indication	SND or AVB	3	82/519	154/526	0.54 [0.43, 0.68]	<0.00001	0.06
	AVB	4	17/190	101/388	0.32 [0.20, 0.53]	<0.00001	
Follow-up	<20 months	3	26/322	81/360	0.38 [0.25, 0.57]	<0.00001	0.39
	≥20 months	5	82/560	217/791	0.46 [0.37, 0.59]	<0.00001	
Sample size	<100 patients	3	9/81	47/114	0.27 [0.14, 0.54]	0.0002	0.14
	≥100 patients	5	99/801	251/1037	0.47 [0.38, 0.58]	<0.00001	
B							
Subgroup		Number of included studies	CSP	RVP	OR (95% CI)	*p*	Group difference
Study design	Prospective studies	3	72/490	137/493	0.41 [0.29, 0.57]	<0.00001	0.16
	Retrospective studies	5	36/392	161/658	0.28 [0.19, 0.42]	<0.00001	
Pacing mode	HBP	3	25/203	106/397	0.31 [0.19, 0.51]	<0.00001	0.60
	LBBP	5	83/679	192/754	0.36 [0.27, 0.48]	<0.00001	
Pacing indication	SND or AVB	3	82/519	154/526	0.42 [0.31, 0.57]	<0.00001	0.12
	AVB	4	17/190	101/388	0.25 [0.14, 0.44]	<0.00001	
Follow-up	<20 months	3	26/322	81/360	0.32 [0.20, 0.51]	<0.00001	0.70
	≥20 months	5	82/560	217/791	0.35 [0.26, 0.48]	<0.00001	
Sample size	<100 patients	3	9/81	47/114	0.18 [0.08, 0.41]	<0.00001	0.10
	≥100 patients	5	99/801	251/1037	0.37 [0.28, 0.49]	<0.00001	

Abbreviations: AVB, atrioventricular block; CI, confidence interval; CSP, conduction system pacing; HBP, His bundle pacing; LBBP, left bundle branch pacing; OR, odds ratio; RVP, right ventricular pacing; RR, risk ratio; SND, sinus node dysfunction.

### 3.3 Publication Bias

**Fig. 4.**
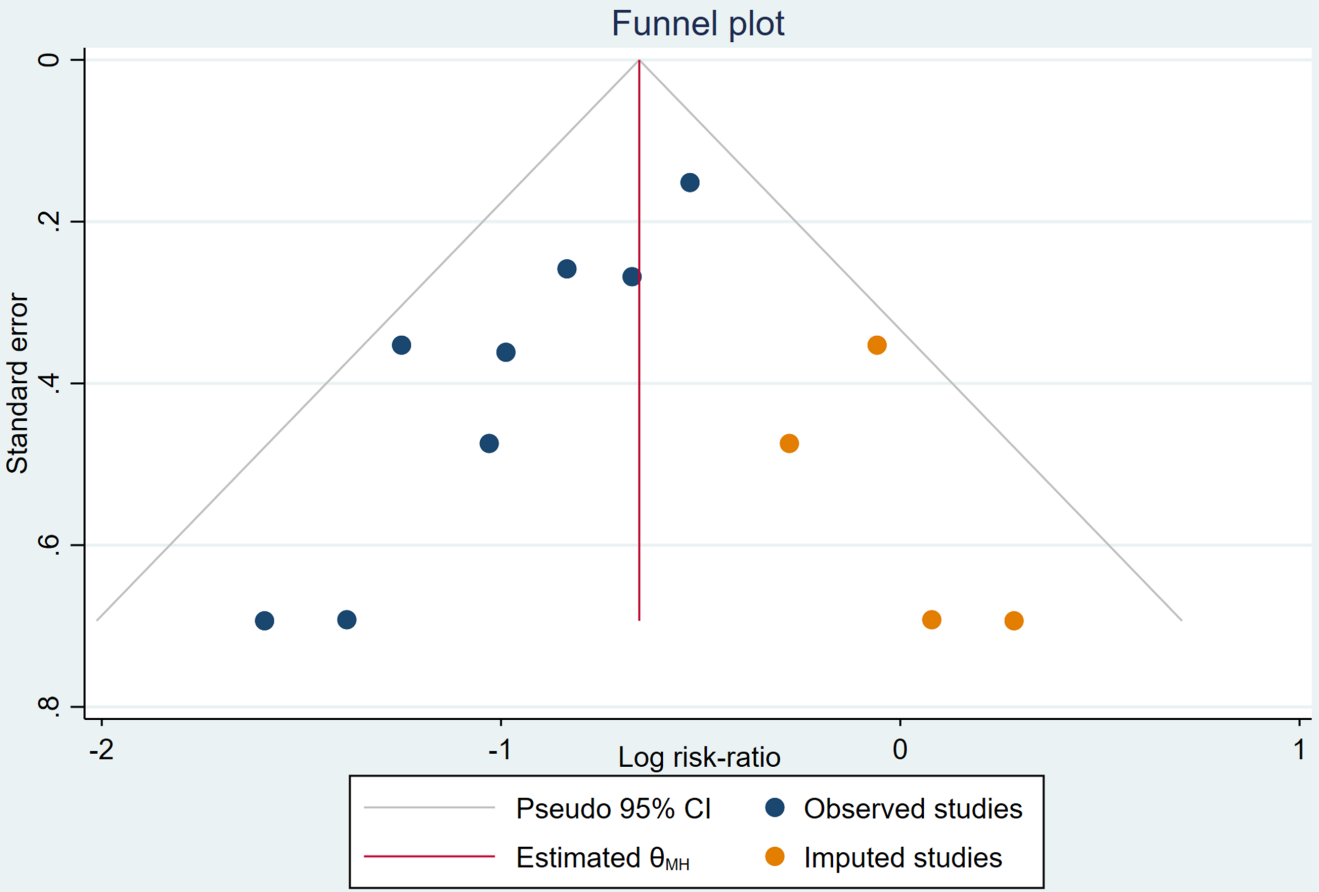
**Funnel plot of random-effects model with trim and fill analysis using RR as effect size, showing original studies (dark blue circles) and imputed studies (dark orange circles)**. RR, risk ratio; CI, confidence interval.

The funnel plot for the new-onset AF was asymmetrical (dark blue circles in Fig. 4 and **Supplementary Fig. 3**) and the *p*-value from the Egger’s test was 0.0125 for RR and 0.0345 for OR, respectively, revealing a significant publication bias. Thus, the trim and fill procedure was executed and 4 missing studies (dark orange circles in Fig. 4 and **Supplementary Fig. 3**) were imputed. After adjustment with imputed studies, the adjusted overall summary effect size was marginally larger than the originally calculated summary effect size but remained significant (RR: 0.51, 95% CI: 0.40–0.64; OR: 0.40, 95% CI: 0.31–0.52, Random-effects model, DerSimonian–Laird method). No evidence of a small study effect was detected, though publication bias might have influenced the observed results.

## 4. Discussion

This is, as far as we know, the most comprehensive meta-analysis to date comparing the risk of new-onset AF between CSP and RVP therapy. The results indicated a significantly reduced incidence of new-onset AF in CSP group, and CSP was correlated to a 49% relative risk reduction when compared with RVP. Furthermore, this advantage of CSP appeared to be particularly evident in patients with a pacing indication of AVB.

New-onset AF was common in patients with PM, and it has been established that a higher VP% is strongly correlated to an increased AF incidence. In a recent meta-analysis including 54 studies comprising a total of 72,784 patients, the overall prevalence of new-onset SCAF was 24.6% in patients without AF history, ranging from 10.1% to 55.7% [20]. In our study, 25.9% of patients in RVP group developed new-onset AF, which was consistent with prior studies. The pathogenesis of VP associated new-onset AF was multifactorial, but the LA remodeling induced by ventricular dyssynchrony during RVP is one of the most important reasons [21, 22]. The non-physiological electrical activation of left ventricle (LV) induced by RVP could impair both the systolic and diastolic function of LV, leading to deleterious hemodynamic changes, including elevated LV end-diastolic pressure and decreased LV systolic volume, which subsequently results in increased LA afterload and LA enlargement. Analogous to long-term RVP, structural and functional changes of LA occurred even after a short-term RVP therapy, which could facilitate the development and progression of AF [6, 23, 24]. Prior clinical investigations have suggested that a higher RVP% is related to adverse clinical outcomes including new-onset AF and pacing-induced cardiomyopathy [1, 9, 10], and each 1% increase in VP% could increase the risk of AF by ~0.7%–1% [9, 25]. Thus, current guidelines recommend minimization of unnecessary VP through programming (I, A) and cardiac physiological pacing in individuals who are anticipated to require a substantial VP% ≥20%~40% (2a or 2b) [1, 26].

As the most physiological pacing strategy in nature, HBP could produce rapid and synchronous ventricular activation through native His-Purkinje system. As a result, compared with RVP, HBP usually yields a narrower paced QRS duration with a superior hemodynamic outcome, and is associated with a reduction in HF hospitalization and new-onset AF [27, 28]. In 2016, Pastore *et al*. [3] observed a reduced non-paroxysmal AF occurrence in the HBP group compared with the RVP group in patients without AF history after an average follow-up of 58.5 months. In another study by Ravi *et al*. [10], HBP exhibited the superior effect in reducing occurrence of new-onset AF compared with traditional RVP, and the benefit of HBP was primarily driven by individuals with VP% ≥20%. These findings of HBP were confirmed by our study which revealed a 60% relative risk reduction (RR: 0.40, 95% CI, 0.26–0.60) for new-onset AF as compared to RVP.

LBBP is one novel physiological pacing modality to achieve the synchronous LV activation and contraction by direct capture of the left conduction system, which offers several advantages in terms of electrical parameters including better pacing threshold and sensed amplitude compared with HBP [29]. LBBP produces a shortened QRS duration and preserves LV electromechanical synchrony, resulting in decreased rates of HF hospitalization and all-cause mortality as compared to RVP [30, 31]. One recent single-center study [32] comprising 19 patients with cardiac resynchronization therapy (CRT) indication compared biventricular pacing-CRT (BiVP-CRT), LBBP-CRP and HBP-CRP by assessing ventricular electrical synchronization and acute hemodynamic response. Though a relatively longer total ventricular activation time due to RV activation delay, LBBP produced a similar left ventricular synchrony and comparable hemodynamic effect when compared with HBP. Existing clinical evidence supports the potential of LBBP as the first-line CSP modality in the future. Zhao *et al*. [5] compared the atrial outcomes between LBBP and RV outflow tract septal pacing in a prospective controlled study enrolling 72 pace-dependent patients, and found that LBBP could increase LA stress, reduce LA pressure and improve LA ejection. Zhang *et al*. [15] observed a small but significant decrease in left atrial diameter in the LBBP group as compared to the RVP group after 1-year follow-up. These beneficial effects of LBBP on atrial remodeling could translate into real clinical benefits in reducing the development and progression of AF, as demonstrated by several observative studies [9, 11, 14, 15, 19]. The subgroup analysis in our study also indicated that LBBP exhibited a comparable effect in reducing new-onset AF compared with HBP, which provided additional clinical evidence for LBBP as the preferred CSP modality in terms of atrial arrhythmia prevention.

Undoubtedly, RVP% is linearly correlated with the risk of AF development, and CSP could reduce the risk of new-onset AF in patients requiring a substantial VP%. In the subgroup analysis based on pacing indications, patients with AVB appeared to benefit more from CSP than those with SND or AVB, probably due to the impact of 33.8% (358/701) patients with SND, who might necessitate less ventricular pacing. One study by Pastore *et al*. [33] indicated that the HBP might be correlated with a reduced risk of persistent AF in SND patients with a long basal PR interval (>180 ms). One retrospective cohort study with 224,814 PM patients evaluated the real-world performance of CSP compared with traditional RVP, and the results indicated that LBBP leads exhibited the comparably excellent pacing parameters [34], without significant difference in complications [30]. The role of CSP, especially LBBP in patients with normal atrioventricular conduction warrants further research considering the likelihood of AVB development in patients with SND during follow-up [35].

For patients with PM, the device could provide us with valuable information about the detection, diagnosis, and pattern of AF through continuous monitoring of intracardiac electrical signals. Patients with SCAF had a 3-fold higher risk of clinical AF development as compared to those without SCAF, and SCAF was also substantially correlated with increased risk of systemic thromboembolism and HF hospitalization [36, 37]. Thus, it is of great clinical relevance to consider new-onset SCAF/AF as an important study endpoint when designing prospective RCTs related to CSP in the future.

### Limitations

This meta-analysis has several limitations. Firstly, all studies enrolled in this meta-analysis were non-randomized, and the majority of them had a limited sample size. Secondly, the definition of new-onset AF was inconsistent among the enrolled studies. Thirdly, there was significant variation in lengths of follow-up, which was of great importance for estimating the study outcome. Fourthly, the study population differed in terms of the pacing indication (SND or AVB) among the enrolled studies, this led to a different VP% among the studies which might influence the incidence of the endpoint event. Fifthly, the impact of different atrial pacing sites on the risk of AF was not examined due to insufficient relevant data. These all might bias the aggregated results of the meta-analysis.

## 5. Conclusion

CSP, including HBP and LBBP, could reduce the incidence of new-onset AF as compared to RVP, and patients with AVB appeared to benefit more from CSP than RVP therapy. Large-scale RCTs are warranted to further evaluate the clinical efficacy of CSP on reducing the occurrence of new-onset AF.

## Availability of Data and Materials

All data generated or analyzed during this study are included in this article. Further inquiries can be directed to the corresponding author.

## Supplementary Material

Supplementary material associated with this article can be found, in the online version, at https://doi.org/10.31083/RCM27921.
