# Supplementary material for: The Risk of New-Onset Atrial Fibrillation in Patients With Conduction System Pacing Versus Right Ventricular Pacing: A Meta-Analysis
Source: Rev Cardiovasc Med. 2025 Apr 18;26(4):27921. doi: 10.31083/RCM27921 (PMC12059758; doi:10.31083/RCM27921)
Supplement: Supplementary file 1 [file 2153-8174-26-4-27921-s1.zip › Supplement materials.docx]

**Supplementary Materials**

**SUPPLEMENTARY NOTES**

**Supplementary Fig. 1** Forest plot of pooled results for new-onset AF between CSP and RVP group using OR as the effect size.

**Supplementary Fig. 2** The leave-one-out sensitivity analysis for causal effect of different pacing modality on new-onset AF using fixed effects model.

**Supplementary Fig. 3** Funnel plot of random-effects model with trim and fill analysis using OR as effect size, showing original studies (dark blue circles) and imputed studies (dark orange circles).

**Supplementary Fig. 1** Forest plot of pooled results for new-onset AF between CSP and RVP group using OR as the effect size. AF, atrial fibrillation; CI, confidence interval; CSP, conduction system pacing; RVP, right ventricular pacing; OR, odds ratio.


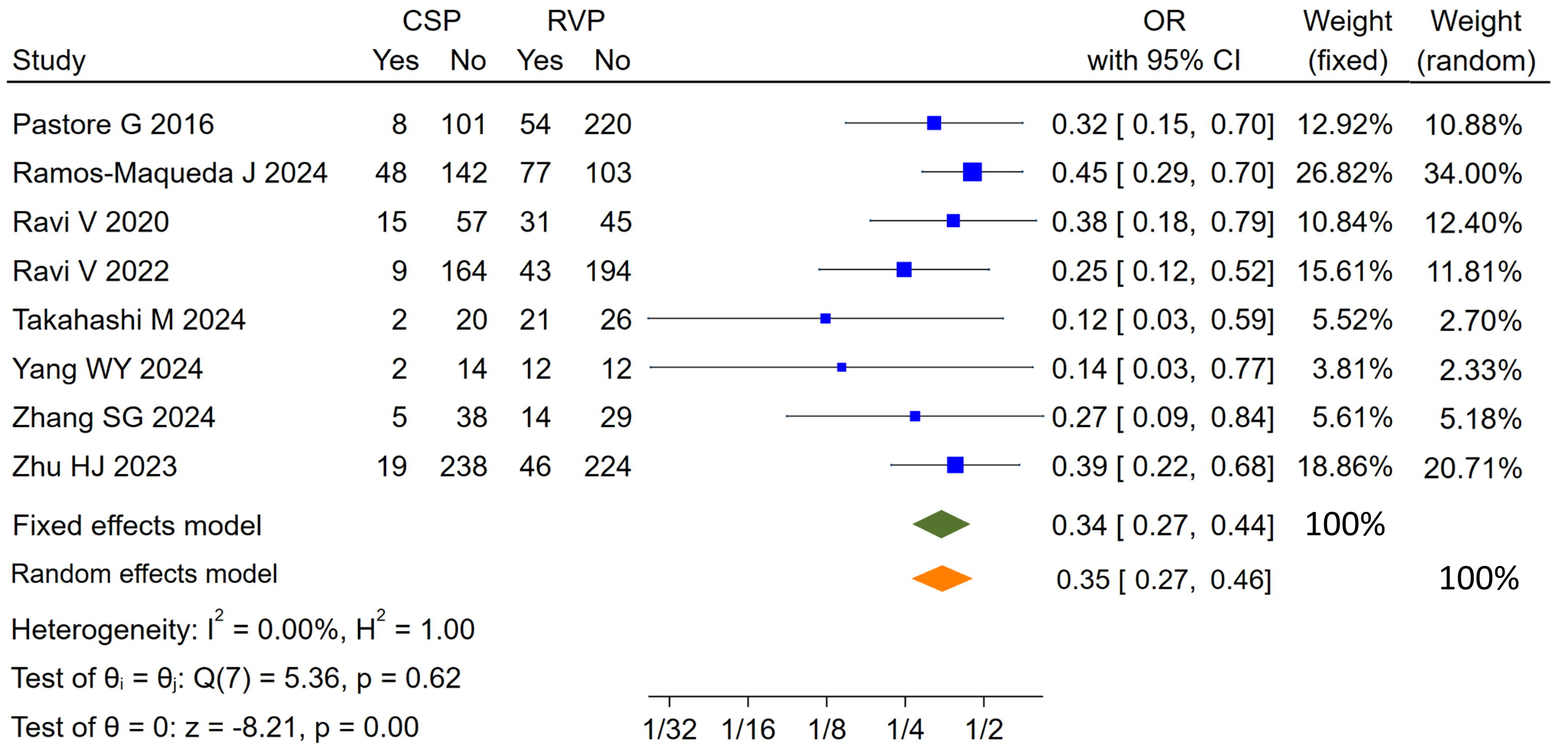


**Supplementary Fig. 2** The leave-one-out sensitivity analysis for causal effect of different pacing modality on new-onset AF using fixed effects model.


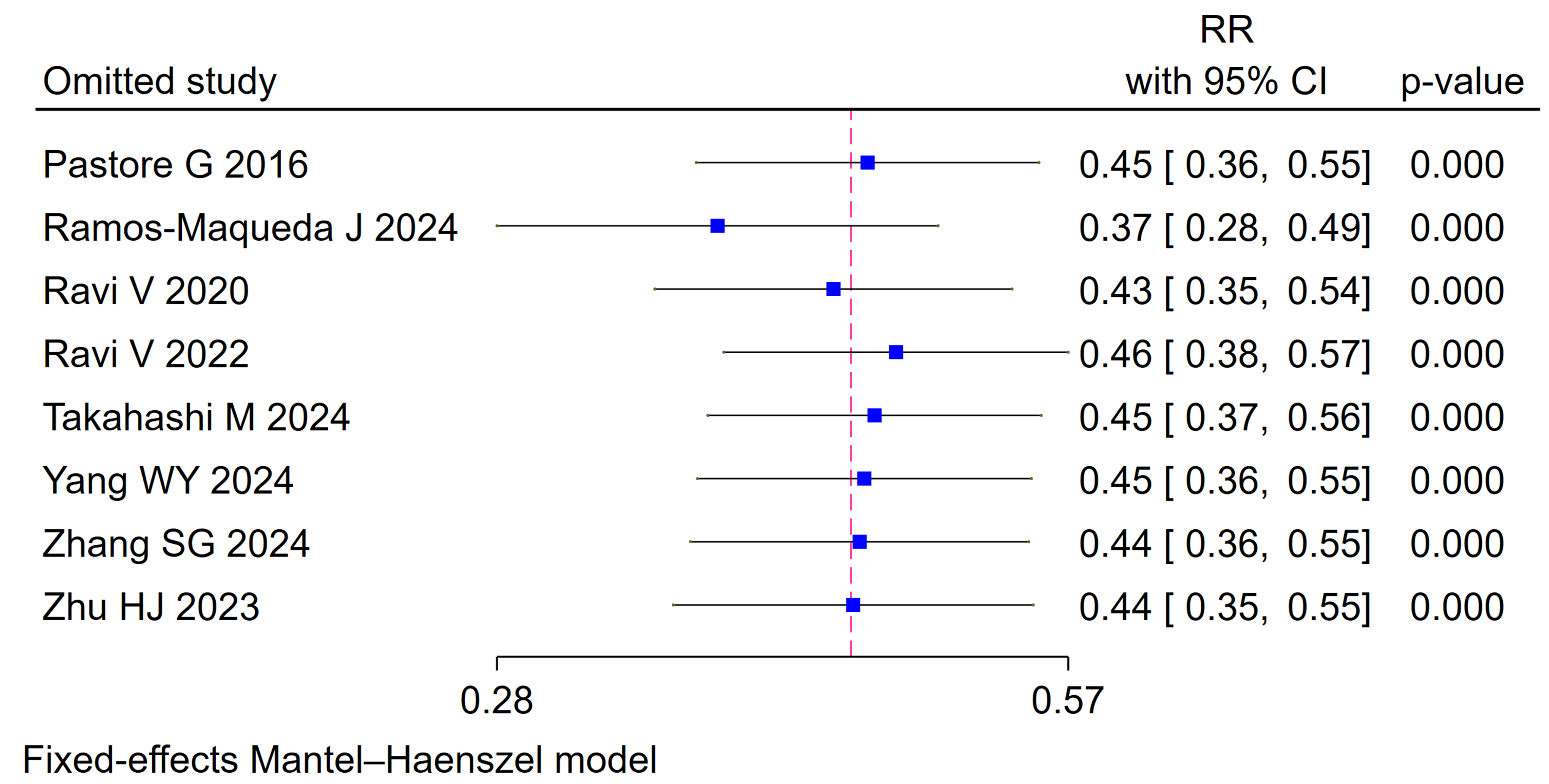


AF, atrial fibrillation; CI, confidence interval; RR, risk ratio.

**Supplementary Fig. 3** Funnel plot of random-effects model with trim and fill analysis using OR as effect size, showing original studies (dark blue circles) and imputed studies (dark orange circles).


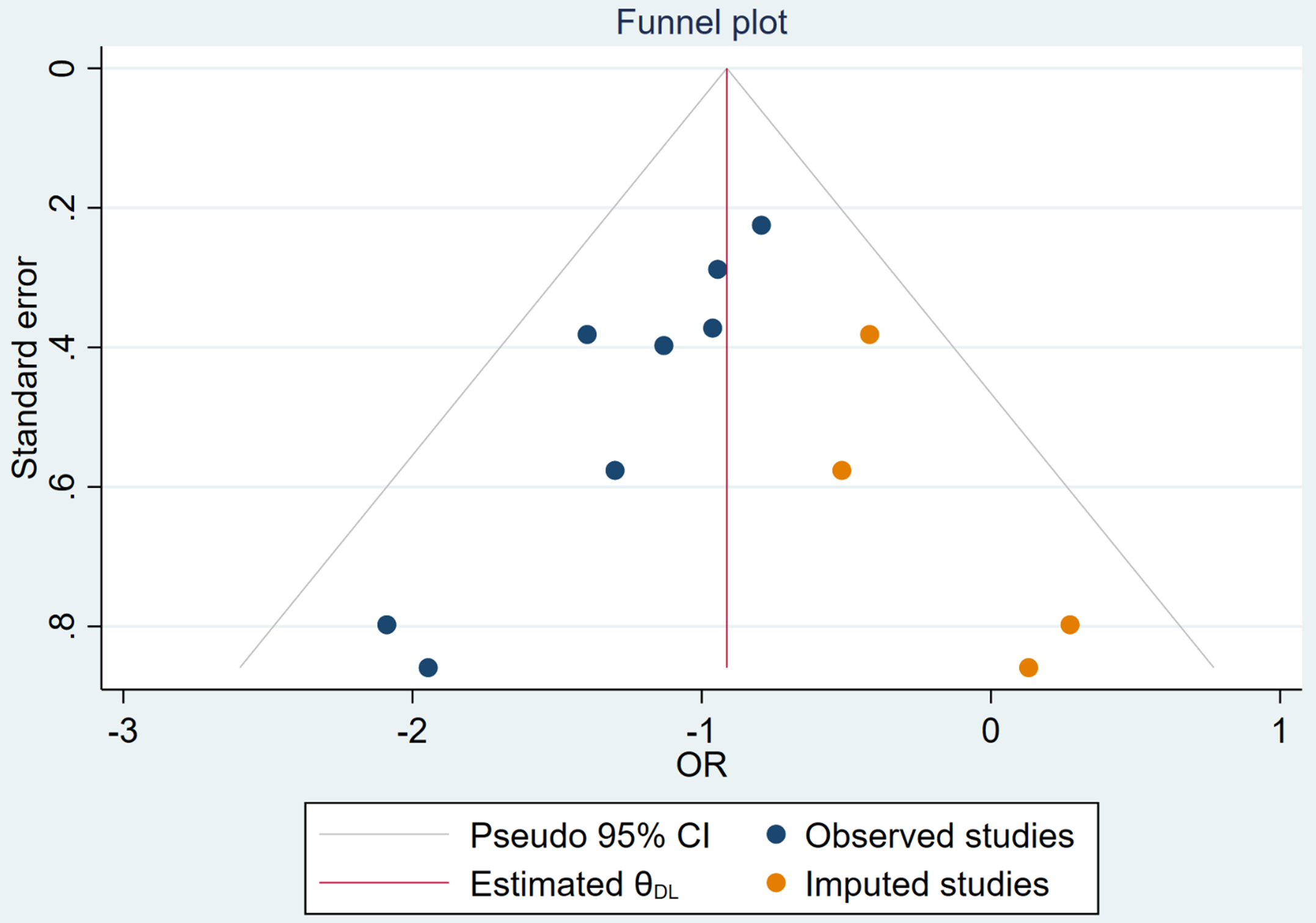


CI, confidence interval; OR, odds ratio.
